# Building Multi-Dimensional Induced Pluripotent Stem Cells-Based Model Platforms to Assess Cardiotoxicity in Cancer Therapies

**DOI:** 10.3389/fphar.2021.607364

**Published:** 2021-02-18

**Authors:** Dilip Thomas, Sushma Shenoy, Nazish Sayed

**Affiliations:** ^1^ Stanford Cardiovascular Institute, Stanford, CA, United States; ^2^ Institute for Stem Cell Biology and Regenerative Medicine, Stanford, CA, United States; ^3^ Division of Vascular Surgery, Department of Surgery, Stanford University School of Medicine, Stanford, CA, United States

**Keywords:** induced pluripotent stem cells, cardiomyocytes, cancer drugs, multicellular crosstalk, 3D platforms, cardio-oncology, drug testing

## Abstract

Cardiovascular disease (CVD) complications have contributed significantly toward poor survival of cancer patients worldwide. These complications that result in myocardial and vascular damage lead to long-term multisystemic disorders. In some patient cohorts, the progression from acute to symptomatic CVD state may be accelerated due to exacerbation of underlying comorbidities such as obesity, diabetes and hypertension. In such situations, cardio-oncologists are often left with a clinical predicament in finding the optimal therapeutic balance to minimize cardiovascular risks and maximize the benefits in treating cancer. Hence, prognostically there is an urgent need for cost-effective, rapid, sensitive and patient-specific screening platform to allow risk-adapted decision making to prevent cancer therapy related cardiotoxicity. In recent years, momentous progress has been made toward the successful derivation of human cardiovascular cells from induced pluripotent stem cells (iPSCs). This technology has not only provided deeper mechanistic insights into basic cardiovascular biology but has also seamlessly integrated within the drug screening and discovery programs for early efficacy and safety evaluation. In this review, we discuss how iPSC-derived cardiovascular cells have been utilized for testing oncotherapeutics to pre-determine patient predisposition to cardiovascular toxicity. Lastly, we highlight the convergence of tissue engineering technologies and precision medicine that can enable patient-specific cardiotoxicity prognosis and treatment on a multi-organ level.

## Introduction

**FIGURE 1:**
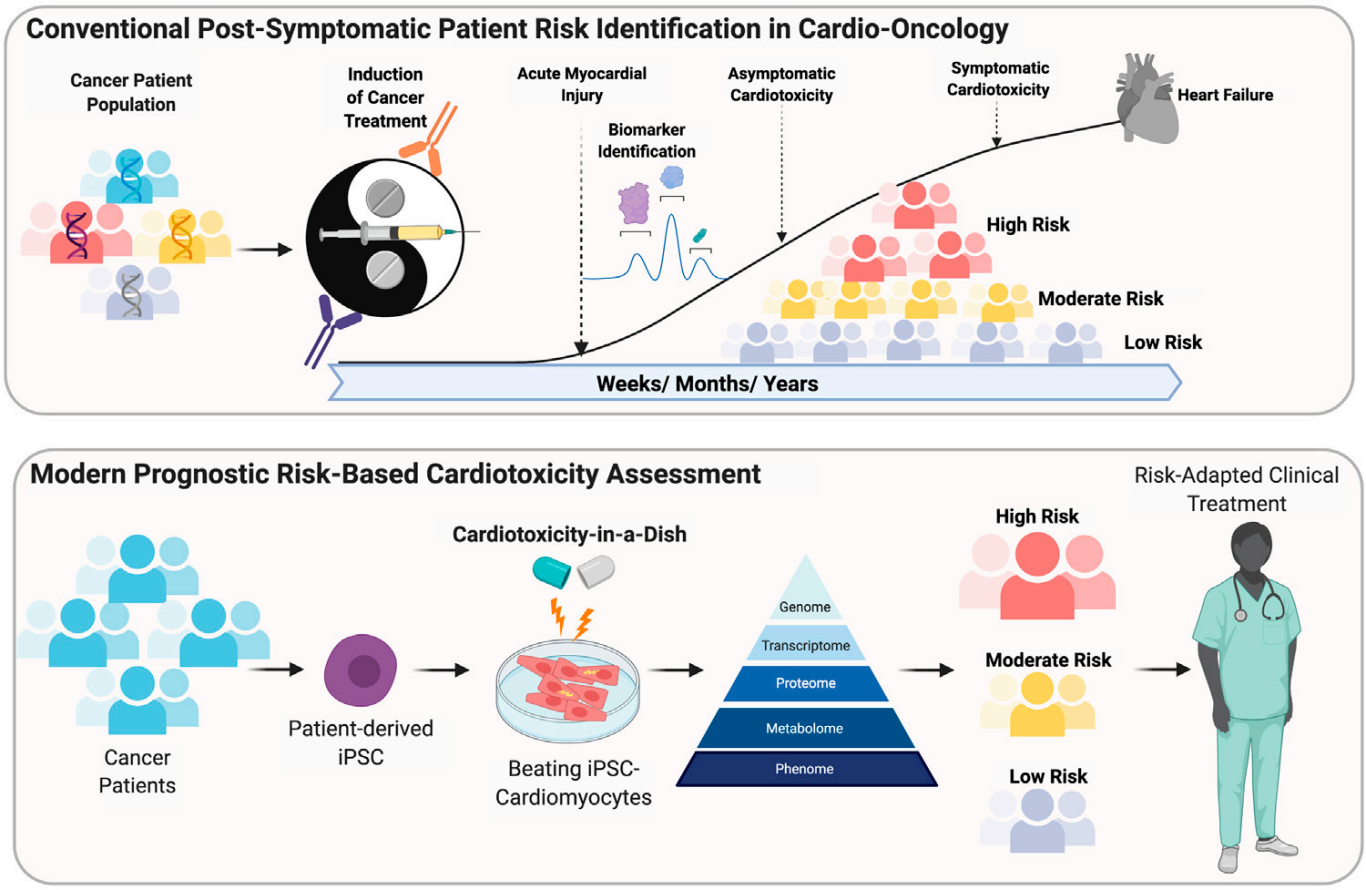
Conventional post-Symptomatic patient risk indentification in Cardio-Oncology. Modern prognostic risk-based cardiotoxicity assessment.

There is a growing burden on the healthcare system with the rise in mortality rate associated with cardiovascular diseases (CVD) and cancer. A 2018 statistics reported ∼17 million deaths globally from CVD and ∼9 million deaths from cancer (Benjamin et al., 2019; Bray et al., 2018; Siegel et al., 2019). By 2040 these figures are expected to double due to a staggering increase in new cases. As of 2019, ∼16 million individuals have been reported as cancer survivors, with nearly two-third of survivors above the age of 60, while 1 in 10 survivors younger than 50 years of age (Miller et al., 2019). It is becoming increasingly evident that cardiotoxicity in cancer patients arise from chemotherapeutic drugs that inadvertently target the heart causing adverse effects such as ventricular systolic and diastolic dysfunction, arrhythmias, pericarditis, myocardial ischemia and heart failure (Figure 1). Indeed, these severe cardiovascular risks further contributes to the mortality rate seen in cancer patients (Sarfati et al., 2016; Miller et al., 2019). Even though, regulatory agencies such as the Food and Drug Administration (FDA) oversee a rigorous review process for new drugs, when it comes to life-threatening diseases such as cancer, the risk-benefit threshold may be higher. In a retrospective study, of all new drug approvals from 1997 to 2016, it was found that 48% had a higher rate of safety-related label changes (Mostaghim et al., 2017). Furthermore, between 2012 and 2017, approximately 95% of all cancer drugs were expedited through the approval process (Hwang et al., 2018). Due to lack of comprehensive testing and recent expedited approval process for many cancer drugs, there are always concerns regarding efficacy and safety. In order to faithfully predict the cardiotoxic side effects of investigational drugs, development of scalable testing platforms *in vitro* is essential. The discovery of human induced pluripotent stem (iPSC) technology has made it possible to regulate organ-specific switches in stem cells to generate any cell type outside the body in a highly controlled artificial environment (Sayed et al., 2016; Sayed and Wu, 2017). In the context of the heart, iPSC-derived cardiomyocytes (iPSC-CMs) has emerged as an attractive testing platform to not only understand basic biology of inherited and non-inherited cardiomyopathies, but also serve as a pharmacological barometer to understand drug-related toxicities and efficacy of new therapeutics (Sayed and Wu, 2017; Sayed et al., 2019; Rhee et al., 2020). In the context of oncotherapeutics, the primary goal is to retard cancerous growth and limit any bystander effects to other cell types of the body that share homologous intra and extracellular targets. Indirect effects of cancer drugs on the heart comprising of multiple cell types may trigger a complex integrated response leading to cardiotoxicity (Gintant et al., 2019). iPSC technology has not only enabled mass production of cardiovascular cell types but also recapitulate disease phenotypes and pharmacological responses. In the recent years, development of standalone engineered tissue systems and high-throughput screening modalities has gained immense interest due to their potential to serve as surrogate clinical trials *in vitro* for safety and efficacy (Fermini et al., 2018). In this review, we summarize the pre-clinical cardio-toxicology studies of chemotherapeutic agents on iPSC-CMs and current limitations associated with the use of iPSC-CMs. Finally, we cover the emerging *in vitro* models that have evolved over the recent decade, offering novel and more predictive alternative for mechanistic assessment of cardiotoxicity and efficacy of oncotherapeutics.

## Induced Pluripotent Stem Cells Cardiomyocytes in Cancer Drug Cardiotoxicity

Assessment of cardiovascular risks using conventional approaches such as non-clinical animal models have been challenging due to striking differences in both biochemical regulation and functional parameters such as beat rate and calcium handling (Sayed et al., 2016). In contrast, human iPSC-CMs share a higher resemblance to their non-human counterparts and offer higher sensitivity and specificity toward cardioactive or cardiotoxic drugs (Grimm et al., 2018). One of the key advantages of using iPSC-CMs is their ability to capture patient-specific drug responses, which may arise from a variety for underlying genetic or metabolic alterations. On a broader scale, iPSC-CMs have shown to exhibit inter-individual variability that enables us to extend our understanding to a larger group of individuals or population for better categorization into responders and non-responders toward a treatment (Burnett et al., 2019).

The potential of iPSC-CMs as an indispensable pre-clinical tool for drug screening assays have already been demonstrated with the Comprehensive *in vitro* Proarrhythmia Assay (CiPA) initiative. CiPA aims to evaluate the proarrhythmic risk of new drugs through a comprehensive mechanistic assessment and validation on human iPSC-CMs. These drug assessments occur in four distinct stages: 1) Characterization of the drug effects on human cardiac currents; 2) In silico reconstruction of the ventricular electrophysiology; 3) modeling the effects on iPSC or embryonic (ES)- derived ventricular cardiomyocytes; and 4) clinical evaluation of cardiac risk. The primary endpoint assay is detection of electrophysiological abnormalities that could be due to changes in repolarising or depolarising ionic currents (iNA, iTo, iCal, iKr, iKs, iK1). Electrophysiological abnormalities are often underreported in several drug induced toxicities but are key early indicators of potential drug-induced adverse effects (Doherty et al., 2013; Sharma et al., 2017). Therefore, systematic characterization and longitudinal assessments are required to understand acute, subacute and chronic toxicities observed in cancer drug-induced cardiac dysfunction. Indeed, several large scale, multi-centre studies have been conducted to detect ionotropic drug effects with well-defined ionic current parameters (Blinova et al., 2018). One of the major limitations of human iPSC-CMs is the immature phenotype that resemble the fetal stage of cardiac development (van den Berg et al., 2015). For example, iPSC-CMs lack morphological, metabolic and electrophysiological maturity when compared to adult cardiomyocytes. To overcome this, several techniques have been developed to enable higher degree of maturation. A comprehensive review of these methods is described elsewhere (Guo and Pu, 2020; Karbassi et al., 2020). Despite these limitations, iPSC-CMs due to their unlimited production capacity are preferred over human heart biopsy samples which are very limited and difficult to maintain *in vitro*. Traditional cell models for oncotherapeutic efficacy testing were created using cancer cell lines. The opportunity to use patient-derived iPSC-CMs and derivatives is a step toward in using the technology to capture the susceptibility of the cancer patients developing cardiotoxicity.

Current cancer drugs that are mainly studied for adverse cardiotoxic events belong to the class of 1) anthracyclines; 2) tyrosine kinase inhibitors; and 3) checkpoint inhibitors. The co-incidence of cardiotoxicity associated with chemotherapeutic drugs was first identified and reported in 1970s (Lefrak et al., 1973; Von Hoff et al., 1979). Since then studies have identified three common mechanisms by which cancer drugs induce cardiotoxicity. These include double stranded DNA breaks, targeting of DNA modifying enzymes to inhibit replication, and blocking of key prosurvival or metabolic signaling pathways.

### Anthracyclines

The most widely used anthracyclines in the clinic are doxorubicin, daunorubicin, epirubicin and idarubicin. These anthracycline class of drugs induce “by-stander” cardiotoxic effects due to non-specificity on targets that are shared between both metabolically active cancerous cells and healthy cardiac cells. Doxorubicin (DOX), in particular have been shown to elicit a dose-dependent effect both in an acute or chronic setting (Doyle et al., 2005) (Sorensen et al., 2003). Indeed, chronic cardiotoxic effects have been reported in adults who were treated with doxorubicin in their childhood (Lipshultz et al., 2005). Multiple studies have been conducted in the past to understand the mechanisms that causes DOX induced cardiotoxicity. For example, mitochondrial oxidative stress caused due to rapid reduction of DOX, disruption of cytochrome enzymes, accumulation of iron and generation of free-radical ions is considered to be one of the modes of toxicity (Davies and Doroshow, 1986; Lebrecht et al., 2003; Ichikawa et al., 2014) (Papadopoulou and Tsiftsoglou, 1993). Similarly, the direct binding of DOX to topoisomerase 2 isoenzymes, which results in double stranded DNA breaks (Lyu et al., 2007; Zhang et al., 2012; Vejpongsa and Yeh, 2014) is considered to be another major factor contributing to cardiotoxicity. Dexrazoxane, an FDA-approved drug has shown to confer cardioprotective effects in DOX treated patients (Swain et al., 1997) by inhibiting the catalytic activity of topoisomerase II β (TOP2B) (Bureš et al., 2017; Jirkovský et al., 2018). Indeed, the iPSC-CM platform has also been employed to study the cardiotoxic effects of DOX. In a recent study, Burridge et al. derived iPSC-CMs from two groups of patients to evaluate the effects of DOX *in vitro* (Burridge et al., 2016). These included patients that developed cardiotoxicity following DOX treatment (DOXTOX) and patients that failed to show cardiotoxic symptoms (DOXCON). iPSC-CMs derived from DOXTOX patients showed poor survival with high oxidative stress and DNA damage when compared to DOXCON iPSC-CMs (Burridge et al., 2016). In another study, iPSC-CMs generated from 45 patients when exposed to varying concentrations of DOX revealed genetic basis for cardiotoxicity, wherein 477 expression variants that modulate signature transcriptomic profile were identified (Knowles et al., 2018). Indeed, genome-wide association studies (GWAS) among patient populations have revealed several significant variants that potentiate direct interaction with the TOP2B promoter region or mediate differential splicing of cardiac troponin gene (Aminkeng et al., 2015; Wang et al., 2016). Such patient stratification and inter-individual variability achieved using iPSC-CMs offer a strong basis for detecting patient-specific responses to cancer therapies based on their genotypic and phenotypic sensitivities.

### Tyrosine Kinase Inhibitors

Human iPSC-CMs have been utilized to screen cardiovascular toxicities associated with tyrosine kinase inhibitors (TKIs) to mirror clinical phenotypes. TKI-associated cardiotoxicities in cancer patients include arrhythmias, myocardial infarction and reduced left ventricular ejection fraction (LVEF) (Force et al., 2007). While TKIs act by blocking tyrosine kinase receptors that stunts cell proliferation, survival and migration, their effects at the cellular level include increased reactive oxygen species (ROS) production, lipid and cholesterol accumulation, and activation of caspases (Doherty et al., 2013; Schwach et al., 2020).

One of the first large-scale studies looking at TKI-induced cardiotoxicity tested 21 FDA approved TKIs on iPSC-CMs derived from 11 healthy and two cancer patients on a high-throughput platform (Sharma et al., 2017). Six TKIs (regorafenib, vemurafenib, nilotinib, crizotinib, sorafenib and vandetanib) exposed to iPSC-CMs were found to exhibit varying degrees of cytotoxic and functional deficit. In particular, three TKIs (sorafenib, regorafenib and ponatinib) were found to be highly cytotoxic causing mitochondrial stress, contractility changes and cell death. In accordance with clinical findings (Lee et al., 2019), two of the TKIs, nilotinib and vandetanib were found to induce arrhythmias at a cellular level. Subsequent studies performed on iPSC-CMs revealed that cardiotoxicity associated with sorafenib is likely due to a metabolic shift from oxidative phosphorylation to glycolysis (Wang H. et al., 2019). Tyrosine kinase activity may also be reduced through blockade of human epidermal growth factor receptor 2 (HER2), a proto-oncogene upregulated mainly in breast cancer. Activation of HER-2 pathway is known to play an important role in cardiac development and drug-induced cardioprotection through endothelial-cardiomyocyte signaling (Lee et al., 1995; Lemmens et al., 2007; Galindo et al., 2014). In an iPSC-CM model, inhibition of HER2 signaling pathway with trastuzumab was shown to cause impairment in contractile and calcium handling properties which were reversed through metabolic modulation with AMP-activated protein kinase (AMPK) (Kitani et al., 2019). Similarly, exogenous treatment with neuregulin-1 (NRG-1) and heparin-binding epidermal growth factor (HB-EGF) has shown to confer cardioprotective effects in iPSC-CMs against DOX, however, co-treatment of DOX and trastuzumab were shown to partially negate the effect of NRG1 (Kurokawa et al., 2018).

### Immunotherapies

Cancer immunotherapy drugs are targeted biologics aimed to reinvigorate the immune cells’ capacity to target and eliminate cancer cells. In chimeric antigen receptor (CAR) T-cell therapy, patient’s T cells are isolated and engineered to express receptors, which upon binding to cancer cells lead to cell death. Sporadic clinical cardiotoxicity has been reported mainly arising from co-expression of tumor targets or antigen cross-reactivity with cardiac proteins (Morgan et al., 2010; Cameron et al., 2013). One of the widely accepted adverse cardiac effects of CAR T-cell therapy is believed to be due to a “cytokine storm” caused by the activation of lymphocytes and the release of inflammatory cytokines. Cytokine storm also known as cytokine release syndrome (CRS) can lower cardiac ejection fraction and cause arrythmias (Neelapu et al., 2018). Immune checkpoint inhibitors (ICIs) are another class of T-cell modulators that prevent T-cells from being turned-off or remain in an anergic state. Several adverse cardiac events have been noted in patients treated with ICIs including myocarditis, vasculitis, electrophysiological abnormalities and arrhythmias (Mahmood et al., 2018; Moslehi et al., 2018). Even though mortality associated with such adverse events are disproportionately high, the incidence of such an event is rare. Target ligands of checkpoint inhibitors such as programmed cell death ligand 1 (PD-L1) expression has been reported in cardiomyocytes (Nishimura et al., 2001). Based on this, it can be speculated that possible underlying mechanisms of cardiotoxicity may be due to 1) homology in antigen expressed on tumor cells and cardiomyocytes, which are recognized by T-cells; 2) T-cell response toward unknown cardiac antigen; and 3) diverse T-cell receptor repertoire with distinct antigen binding on both tumor cell and cardiomyocytes followed by initiation of an effector response (Brown et al., 2020). Co-culture studies using iPSC-derived cardiovascular cells and primary T-cells or exposure to patient’s serum may reveal more insights into active biomarkers or non-canonical molecular interactions that result in myocardial toxicities in these patients.

## Addressing Functional and Cellular Heterogeneity in Cardiomyocytes Derived From Induced Pluripotent Stem Cells Sources

hiPSC-CMs have emerged as one of the important predictive tools is due to its ability to retain genetic identity of the patient for disease modeling and discovery of personalized medicine. However, somatic cell sources for pluripotency induction, differences in cardiomyocyte differentiation protocols and heterogeneity in myocyte composition can lead to inconsistencies in the outcomes of pharmacological studies. Several studies have indicated that differences in genetic background and iPSC-CM derivation methodologies can influence epigenetic landscape and consequently gene expression. For example, iPSCs derived from cardiac progenitor cells are shown to have higher cardiac differentiation potential compared to non-cardiac sources (Sanchez-Freire et al., 2014; Meraviglia et al., 2016). This suggests that although reprogramming erases most epigenetic modifications, some tissue-specific signatures may remain unaltered and thereby influence cardiac differentiation potential (Kim et al., 2011; Rouhani et al., 2014). From a drug testing point-of-view, epigenetic variations may alter cellular responses to drugs, overriding the phenotypic response. Such changes can be overcome by adopting uniform reprogramming methodology to reduce epigenetic alterations (Bar and Benvenisty, 2019). In addition to higher reproducibility, iPSC-CM drug responses can be validated by generation of artificial intelligence (AI) algorithms that can systematically compare endpoint measurements such as action potential (AP) or calcium handling parameters to extrapolate experimental outcome for a given class of drug (Juhola et al., 2018; Kernik et al., 2020). However, the full potential of such tools can only be exploited with data generated using iPSC-CMs of comparable quality. Integration of several such comprehensive data types will help distinguish the complex causal relationships from the noise introduced due to the quality of the cells.

To date several key optimizations in cardiac differentiation have been explored to derive iPSC-CMs using simple, cost-effective and xeno-free methods (Lian et al., 2012; Burridge et al., 2014). Despite these refinements, current differentiation protocols provide satisfactory yield for small-scale use. Furthermore, there is considerable variability in obtaining highly pure population of ventricular, atrial or pacemaker subtypes. To minimize batch-to-batch variability, reproducibility and functional quality of the cardiomyocytes generated, small-scale protocols need to be robustly tested in large scale culture devices and bioreactors. Currently, static two dimensional (2D) multi-layer flasks and dynamic three-dimensional (3D) microcarrier adherent systems are able to generate 1.5–2.8 billion cardiomyocytes in a single bioprocess (Correia et al., 2014; Tohyama et al., 2017). A recent report also demonstrated that iPSC-CMs can be expanded to a hundred-fold through inhibition of glycogen synthase kinase-3β (GSK-3β) pathway during early stages of cardiac differentiation. These cells obtained can be purified using non-genetic methods such as metabolic selection with lactate-containing medium or a dye to label mitochondria that occupy ∼40% of the CM volume (Hattori et al., 2010; Tohyama et al., 2013).

With regard to function, in adult cardiomyocytes mature structural features are intertwined with functional regulation. Unlike adult cardiomyocytes, neonatal or iPSC cardiomyocytes do not show rectangular morphology, exhibit spontaneous generation of AP, have lower density of mitochondria and higher dependence on glycolytic metabolism over fatty acid oxidation. Several recently developed techniques help iPSC-CM maturation through metabolic supplementation (Parikh et al., 2017; Feyen et al., 2020), incremental pacing (Chan et al., 2013) and culture substrate modifications to promote higher contraction forces (Pandey et al., 2018). Currently, these culture procedures from somatic reprogramming to obtaining functionally mature iPSC-CMs require over a month’s time, which may limit their use for point-of-care testing. Therefore, utilization of a single platform that enables rapid generation of functionally mature iPSC-CMs from the donor cells will accelerate the drug testing timeframe.

## Building Physiologically Relevant *In Vitro* Cardiac Models

**FIGURE 2:**
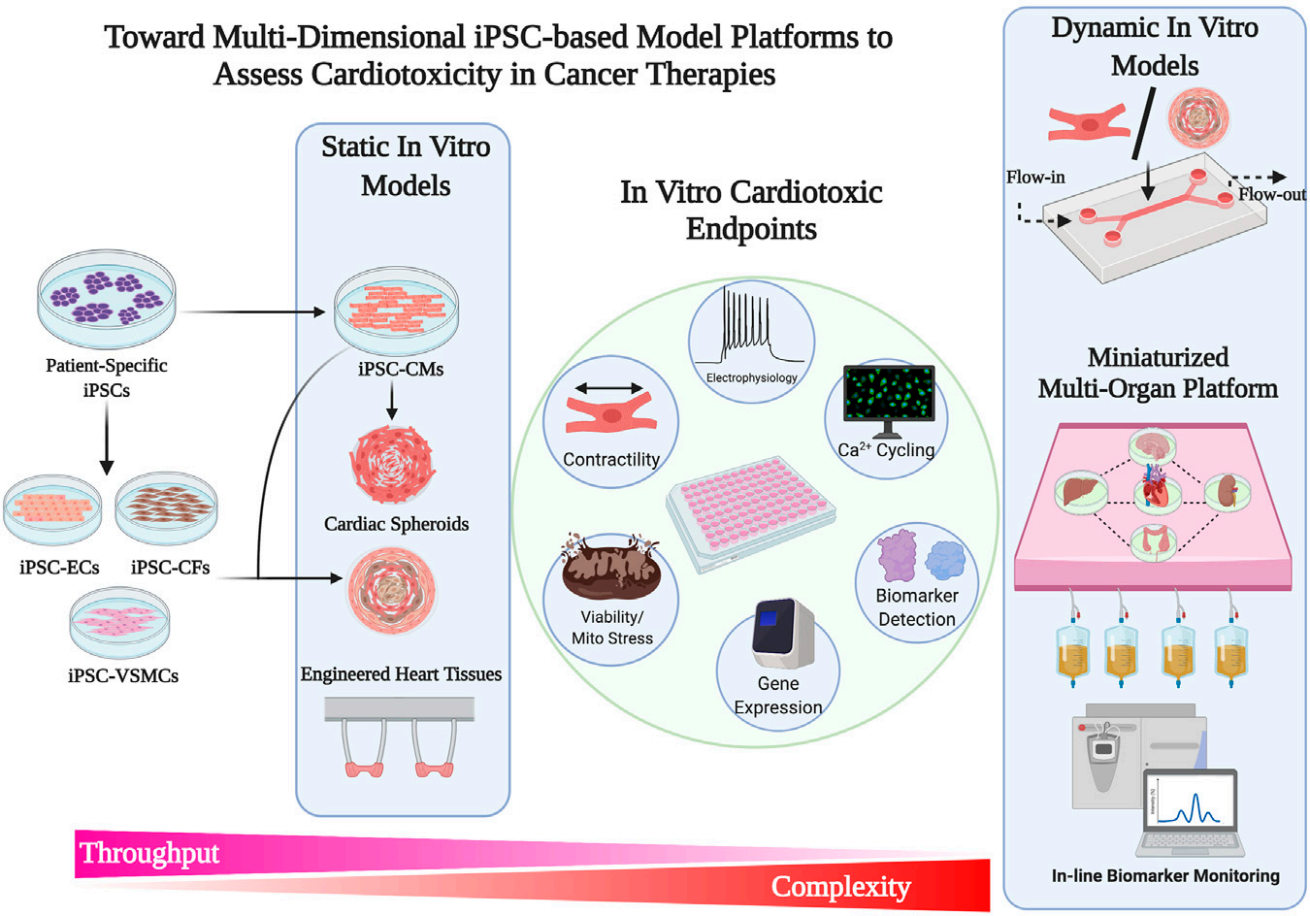
Toward multi-dimensional iPSC-based model platform to access Cardiotoxicity in Cancer therapies.

Current drug discoveries and therapeutic testing takes place on conventional 2D tissue culture platforms. However, there are several limitations with using platforms that are non-physiological in a reductionist manner. 2D planar culture platforms offer flexibility and ease of use, however, fail to capture the structural complexities that are seen in 3D tissues and organ systems. These 3D tissues develop through gradients of biochemical and mechanical signaling, and multicellular crosstalk that contribute to the maintenance of tissue homeostasis. In a biological context, 2D platform offers the least resistance at a single-cell level wherein cellular turnover, metabolism, and protein synthesis occur in an accelerated manner. In contrast, in a 3D tissue-like architecture these processes occur in concert with changes in the local extracellular environment, receiving feedback responses from a multitude of cells that are tightly packed in a small volume (Baker and Chen, 2012; Thomas et al., 2018; Thomas et al., 2020). With the recent advancements in bioengineering tools, 3D culture systems with varying degrees of complexities have gained significant traction in the field of drug testing and drug discovery (Figure 2). Indeed, 3D cultures of iPSC-CMs in the form of engineered myocardium have been shown to enhance physiological hypertrophy, improve maturity, and enhance drug response (Karbassi et al., 2020).

### Three Dimensional Cardiac Constructs for Drug Testing

iPSC-CMs have been used to generate 3D myocardium as self-assembled, scaffold-free, spontaneously beating clusters referred as cardiac spheroids. Alternatively, iPSC-CMs can also be embedded in natural or synthetic extracellular matrix (ECM) in form of engineered heart tissues (EHTs) that allow anisotropic tissue-like orientation and cellular alignment. iPSC-CMs in both scaffold-free cardiac spheroids and matrix assembled tissues have shown to exhibit more mature characteristics when compared to 2D cultures (Hoang et al., 2018). Maturity training in 3D cultures are conferred using biophysical stimulation with passive stretch and electromechanical conditioning (Ronaldson-Bouchard et al., 2018); whereas biochemical stimulation is induced by metabolic and hormonal programming (Parikh et al., 2017). Obtaining a “near” physiological maturation through such techniques can significantly improve drug responses. From a disease modeling perspective, EHTs fabricated from iPSC-CMs manifest a more clinically relevant phenotype in familial arrhythmogenic syndromes compared to iPSC-CMs at a single-cell level (Goldfracht et al., 2019). In two recent studies, EHTs derived from iPSC-CMs demonstrated a high force-frequency relationship and physiological response to ionotropic and chronotropic drugs (Mannhardt et al., 2017; Ronaldson-Bouchard et al., 2018).

As a platform technology for high-throughput screening, iPSC-CMs have been assembled into EHTs on a 24-well (Hansen et al., 2010) and 96-well formats (Mills et al., 2019) that are amenable to rapid screening of drugs to test safety and efficacy. For instance, EHTs derived from rat neonatal cardiomyocytes when treated with gefitinib (10 µM), lapatinib (150 µM), sunitinib (10 µM), imatinib (100 µM) and sorafenib (100 µM) showed significantly reduced tissue contractility; whereas vandetanib and lestaurtinib showed a dose-dependent decline in function (Jacob et al., 2016). In another study, EHTs derived from human iPSC-CMs were fabricated around soft and stiff posts to mimic pre-load and afterload conditions (Truitt et al., 2018). These EHTs when exposed to clinically relevant concentration (1–10 μmol/L) of sunitinib showed a significant increase in caspase-induced cardiotoxicity due to afterload. Here it is important to note that 3D platforms due to their higher tissue organization and slower diffusion kinetics may impart higher drug sensitivity thresholds similar to *in vivo*, unlike 2D culture systems. Furthermore, for the assessment of potential drug-induced toxicity, model systems that are relevant to human cardiac physiology and diseases may be more suitable over other species due to inherent differences in function and drug sensitivity. An overview of 2D and 3D stem cell derived cardiomyocyte platforms used for chemotherapeutic testing on cardiovascular cells is summarized in Table 1.

**TABLE 1:** A summary of in vitro stem cell-based cardiac models for cancer drug testing.

Format	Drug class	Drug	Concentration	Cell type	Cell number	Cardiomyo-cyte age	Effect	Ref
2D	Anthracycline	Doxorubicin	1–10 μM	hiPSC-CM (mixture ventricular and atrial)	0.004–0.33^×10^ 6/cm^2^	3–4.5 weeks	Lower amplitude, beat frequency and decreased FPD	Burridge et al. (2016), Maillet et al. (2016)
2D	Anthracycline/TKI	Doxorubicin/sunitinib/crizotinib	0.1–100 μM	hiPSC-CMs/iCell®	0.08^×10^ 6/cm^2^	-	Reduced cell viability, mitochondrial integrity, Increased cAMP formation and lower beat frequency	Grimm et al. (2015)
2D	Anthracycline	Doxorubicin	1–10 μmol/L	hiPSC-CM	0.26^×10^ 6/cm^2^	3.5–8 weeks	Dose and cell-age dependent apoptosis and ROS production. DNA damage via TOP2A	Cui et al. (2019)
2D	Anthracycline	Doxorubicin	0.05–0.45 μM	Cellartis^®^ Pure hES-CM	0.2^×10^ 6/cm^2^	-	Increased cardiac Troponin T release at day 2, Increased expression of apoptosis and p53 signalling pathways	Holmgren et al. (2015)
2D	TKI	Vandetanib	0.1–1 μM	hiPSC-CM (mixture ventricular, atrial and nodal)	0.003^×10^ 6/cm^2^	4.5 weeks	Prolongation of repolarization and arrhythmia	Blinova et al. (2018)
2D	TKI	Sunitinib	0.3–10 μM	hiPSC-CM (mixture ventricular, atrialand nodal)	0.002^×10^ 6/cm^2^	4.5 weeks	FPD Prolongation and early afterdepolarization (EAD)	Nozaki et al. (2017)
2D	TKI	*niolotinib, Vandetanib (*21 TKIs tested)	0.1–100 μM	hiPSC-CM, hiPSC-ECs, hiPSC-CFs	0.44^×10^ 6/cm^2^	4–5 weeks	Prolonged FPD, alterations in CM contractility and calcium handling	Sharma et al. (2017)
2D	TKI	Lapatinib, Sorafenib, Erlotinib and Sunitinib	0.01–10 μM	Cor.4U hiPSCCMs	0.07^×10^ 6/cm^2^	-	Reduced mitochondrial potential and respiration, decreased troponin expression	Wang H. et al. (2019)
2D	Her2 monoclonalantibody/TKI	Trastuzumab/Lapatinib	100 μg/ml/2 μM	hiPSC-CMs/iCell^®^	0.06^×10^ 6/cm^2^	-	Downregulation of ERBB2 with reduced media glucose levels after trastuzumab treatment. Both trastuzumab and lapatinib downregulated expression of PDK and upregulated PHLDA1	Necela et al. (2017)
2D	Her2 monoclonal antibody/Anthracycline	Trastuzumab - Doxorubicin co-treatment	1 μM	hiPSC-CMs/iCell^®^	0.06^×10^ 6/cm^2^	-	Concentration dependent reduction in cell impedance and ATP	Eldridge et al. (2014)
2D	Her2 monoclonal antibody/Anthracycline	Trastuzumab/Doxorubicin	0.1–1μM/0.05–0.1 μM	hiPSC-CMs	0.12^×10^ 6/cm^2^	∼2.5–3 weeks	Dose-dependent reduction in contractility, calcium handing and mitochondrial stress	Kitani et al. (2019)
2D	Her2 monoclonal antibody/Anthracycline	Trastuzumab/Doxorubicin	1 μM/10 μM	hiPSC-CMs and primary ECs	0.16^×10^ 6/cm^2^	∼4–5 weeks	Increased LDH release in doxorubicin only treatment compared to doxorubicin, trastuzumab and neuregulin-1	Kurokawa et al. (2018)
2D	Anthracycline	Doxorubicin	5 μM	Multi-organ on a chip in monolayer (hiPSC-CMs/iCell^®^,human HepG2 hepatocellular carcinoma cells , human skeletal muscle, hiPSC cortical neurons	0.1–0.16^×10^ 6/cm^2^	-	Concurrent dose-dependent toxicity in hepatocytes and cardiomyocytes, no action potential changes in neurons	Oleaga et al. (2016)
3D	Anthracycline	Doxorubicin	3.5–100 μM	hiPSC-derived cardiac bodies on a chip	-	-	Dose-dependent decline in beating frequency	Bergström et al. (2015)
3D	Anthracycline	Doxorubicin	1–40 μM	Cardiac spheroids composed of iPSC-CMs, iPSC-CFs and primary ECs	3,000–6,000/construct	-	Dose-dependent reduction in cell viability and increase in endothelial nitric oxide	Polonchuk et al. (2017)
3D	Anthracycline	Doxorubicin	0.5–20 μM	Cardiac spheroids composed of iPSC-CMs and fetal CFs	5,000/construct	-	Contractile dysfunction and irregular contractions with pacing	Beaucham p et al., 2020
3D	Anthracycline/TKI	Doxorubicin/sunitinib	30μM/100 μM	Cardiac spheroids composed of hiPSC-CMs/iCellR , primary human microvascular ECs, and human primary CFs	500/construct	-	Reduced mitochondrial membrane potential and ATP. Elevated cTnI, CK-MB and FABP-3 after treatment. Reduced expression of cTnI, vimentin and α-actinin	Archer et al. (2018)
3D	TKI	Sunitinib	1.10 μM	Engineered Heart Tissue composed of hiPSC-CM and hMSC	-	2.5–4 weeks	Load induced increase in caspase3/7	Truitt et al. (2018)
3D	Anthracycline	Doxorubicin	1–1000 nM	Cardiac spheroids composed of hiPSC-CMs, primary CFs and primary microvascular ECs	100,000–5 × 106/construct	-	Decline in beat rate with increase in concentration and reduction vascular capilaries	Amano et al. (2016)
3D	Anthracycline	Doxorubicin	0.1–10 μM	Cardiac spheroids composed of hiPSC-CMs	5 × 105/construct	-	Concentration-dependent reduction in cell viability	Takeda et al. (2018)
3D	Anthracycline/Alkylating agent	Doxorubicin/Oxaliplatin	0.1–10 μM/0.1–50 μM	Colon carcinoma SW620, hiPSCCM and hiPSCECs on a chip	0.3–1 × 108/ml gel	4–6.5 weeks	Dose-dependent decline in viability, beat rate, conduction velocity	Weng et al. (2020)
3D	Anthracycline	Doxorubicin	5, 25 μM	Human HepG2 hepatocellular carcinoma cells and hiPSC-CM organoids on a chip	1 × 107/ml gel	-	Reduced viability, decrease in albumin secretion, increase in alpha glutathione s-transferase from hepatocytes, increase in creatine kinase MB in cardiomyocytes and decline in beat rate	Zhang et al. (2017)

At a cellular level, decreased contractile function due to mutational cardiac disorder or drug-induced toxicity is often associated with cytoskeletal disarray and poor structural integrity. These changes influence electromechanical coupling and biomechanical properties both at the cellular as well as tissue level. A combination of atomic force microscopy (AFM) coupled with MEA platform could be used to measure sensitive biological changes in topography and force (Caluori et al., 2019).

In an adult human heart, over 60% of the tissue comprises of non-myocytes, namely endothelial cells, vascular stromal cells, cardiac fibroblasts and a small fraction of immune cells (Pinto et al., 2016). Crosstalk between myocytes and non-myocytes are essential for maintaining a physiological balance and cardiovascular tone. Endothelial cells support cardiac metabolism, contractility and survival (Brutsaert, 2003; Sayed et al., 2020); whereas fibroblast play a key role in metabolic, structural, electrical and mechanical maturation (Giacomelli et al., 2017; Giacomelli et al., 2020). Therefore, it is intuitive that these major non-myocyte cell populations may play a significant role in disease and drug-related toxicities. Formation of multi-cell type scaffold-free cardiac clusters or spheroids offer higher surface area to volume ratio, and therefore have higher rates of diffusion and mass transport. However, unlike EHTs due to lack of structural guidance, cytoskeletal alignment is not observed. One of the benefits of using cardiac spheroids is their smaller size would allow easy integration into “tissue chips” or multi-well formats for high content imaging and toxicity profiling. Cardiac spheroids fabricated in 384-well format composed of iPSC-CMs, primary endothelial cells and fibroblasts have been utilized to demonstrate DOX-induced cytotoxicity at a concentration greater than 5 µM and sunitinib-induced cytotoxicity at a concentration greater than 10 µM (Archer et al., 2018). Similar studies have been performed to demonstrate DOX-mediated cytotoxicity in multicellular cardiac spheroids (Amano et al., 2016; Polonchuk et al., 2017; Beauchamp et al., 2020). These 3D *in vitro* cardiac models although offer very valuable insights in mimicking tissue complexities and multicellular interaction, they do not fully capture the dynamic pharmacokinetics and pharmacodynamics in volved in drug metabolism and absorption. Hence scaled-down models of interconnected multi-organ systems may provide additional insights that are not currently offered by static 3D *in vitro* models.

### Microphysiological Devices: A New Age of Cardiotoxicity Testing

For research purposes, several ECM substrates are commercially available. Once a 3D framework is established, it is imperative to consider incorporation of modular interconnected multi-organ tissue assemblies on a single platform. 3D printing technologies and microfabrication of tissue chip devices are aimed toward engineering of scaffolds in spatially defined chambers for cell seeding and tissue assembly. Moreover, incorporation of channels in these devices provide essential nutrients and growth factors that help recreate a dynamic microenvironment that is far superior than static organotypic *in vitro* models. Furthermore, integrated platforms such as these combined with biosensors can enable continuous monitoring over long period of time. A fully integrated multi-organ platform developed by Zhang et al. demonstrated drug-induced organ toxicity using a liver-and-heart-on-a-chip comprising of human iPSC-derived cardiac spheroids and human primary hepatocyte-derived liver organoids (Zhang et al., 2017). The authors were able to model the conversion of capecitabine by the liver organoids to 5-fluorouracil (5-FU), a well-known cardiotoxic chemotherapeutic, which caused pronounced toxicity in cardiac spheroids. On the same platform, by replacing the lung organoids with hepatocellular carcinoma cells (HepG2/C3A), the authors demonstrated cytotoxic effect of DOX on cancer cells at 5 and 10 µM. The consequent toxicity-induced cell death in cardiac spheroids was measured using release of cardiac creatine kinase (CK-MB). A similar approach using human primary cardiomyocytes and HepG2 cells was demonstrated using DOX as a model drug (Kamei et al., 2017). In this study, liver cells were shown to generate doxorubicinol, a DOX metabolite, that is responsible for cytotoxicity rather than DOX itself in primary human cardiomyocytes.

Tissue chips with robust *in situ* monitoring of metabolic and functional parameters can be used to model both acute and chronic toxicity to drugs. Currently, there are several limitations with regard to the variability in cell sources used in these devices, such as the use of biopsies or immortalized cell lines. In order to achieve higher resemblance to patient phenotype, the organ specific cells used on the chips should be derived from patient’s iPSCs. Secondly, assembly of cells into more organized hierarchical structures over random cell clusters may provide a deeper understanding of the tissue microenvironment (Thomas et al., 2018). Finally, significant efforts must be directed toward development of universal culture medium, which can be perfused through microchannels to support optimal function and maturation of tissues in these devices. The challenge of developing a universal medium can be overcome by segregated organ specific media reservoirs with an external loop for exchange of media metabolites. In the future, modular organ systems can be further simplified by adopting cartridge-based assemblies for testing synergistic effects of drug toxicity on a multi-organ platform.

## Integrative Panomic Technologies for Mechanistic Insights into Cardiovascular Toxicity

High-throughput quantitative multiplex assays provide a comprehensive understanding of signal transduction networks and molecular signatures that emanate from oncogenes. Similarly, they can detect potential pathways that are activated in other somatic cells that contribute toward organ-specific or systemic toxicity. *In situ* integration of tissue chip devices with online analytical tools for quantitative gene expression or proteomic profiling can be powerful in deciphering intra and inter-cellular communications. Drug metabolism studies in liver slices on a chip, infused with substrates or inhibitors have been coupled with high-performance liquid chromatography with UV detection (HPLC-UV) for metabolite analyses. This on-line rapid detection of drug metabolites and inhibitors can be utilized to identify new biomarkers that can predict cardiotoxicities (van Midwoud et al., 2011). A key advantage of these platforms is their data acquisition and interpretation, which can be integrated within the system to allow feedback-dependent real-time changes in control parameters. In contrast, traditional approaches for transcriptomic profiling limits the understanding of cellular heterogeneity.

Advances in the latest single-cell analyses tools allow profiling of transcriptomic changes at a higher resolution (Tang et al., 2009). The technology since its inception has further evolved with a combination of spatial information, wherein tissue sections attached to a transcriptomic slide with barcoded primers bind and capture mRNAs from the adjacent cells or tissues (Ståhl et al., 2016). For example, using single-cell RNA sequencing (scRNA-seq), a recent study identified a novel gene expression signature (matrix Gla protein) in breast cancer patients treated with trastuzumab that can serve as a prognostic marker for long-term survival. Furthermore, the study revealed expression of 48 genes specifically associated with cardiotoxicity, which can serve as potential biomarkers for trastuzumab-induced cardiotoxicity (Wang J. et al., 2019). scRNA-seq has also been implicated in understanding the effects of novel immune based checkpoint inhibitors. A recent study employed a computational approach to identify interactions based on putative ligand-receptor expression in breast cancer tumor and immune cells. The study showed that radiosensitivity of the tumor played a key role in increased PDL-1 expression, resulting in immune cell inactivation due to their interaction via programmed cell death protein 1 (PD-1) (Jang et al., 2020). Such key findings in a clinical setting can be extremely valuable to devise combined therapeutic strategies followed by subsequent monitoring for cardiac immune-related adverse events (irAEs). On a 3D level, single cell transcriptomics can be applied to help resolve spatial transcriptomics while maintaining structural resolution at a tissue-level. Indeed, in a seminal study by Wang et al., the authors used STARmap approach to identify 23 distinct cell clusters from over 30,000 cells across six layers of mouse visual cortex (Wang et al., 2018). Most recently, a spatiotemporal transcriptomic atlas of a developing human heart was established to reveal developmental dynamics during cardiogenesis (Asp et al., 2019). Furthermore, power trajectory inference analyses can be applied to such large-scale transcriptomic data to understand the progression of each cell as a function of time (Saelens et al., 2019). Lastly, large-scale patient multi-omics data can be integrated into a systems pharmacogenomics approach to identify actionable biomarkers that can reduce cardiovascular risks in cancer patients and survivors (Menche et al., 2015).

## Conclusion

Cardiovascular diseases and several types of cancers have common risk factors and share intricately related pathogenesis that are linked via common cellular pathways. Therefore, the negative consequences of cancer drugs on the cardiovascular system is inevitable. Although conventional toxicology studies in animal models have translational value, they can be cost ineffective and time intensive. As an alternative, iPSC technology can be harnessed to provide a comprehensive phenotypic and genotypic trait that can be used clinically to make therapeutic decisions. Derivation of cardiovascular cells from cancer patients can be utilized to characterize unique functional and genomic signatures that may influence potential adverse reactions to clinically approved drugs. However, with regard to cell maturity there are several milestones that still needs to be reached for minimizing the batch-to-batch variability and the time taken to generate cell or tissue models for a timely and accurate pharmacological prediction. Creation of multiple tissue types using iPSCs can be tested alongside tumors from the same patient in a microphysiological tissue chip devices to tease the mechanistic effects of onocotherapeutics on the body. Such “patient-on-a-chip” models will aid in stratification of cancer patients based on the sensitivity and efficacy of oncotherapeutics (Low et al., 2020). Currently, there are several challenges associated with using multi-organ tissue systems routinely. These are mainly due to complex fabrication process, inadequate physiological fluid flow, unavailability of universal medium for different organ types. Concerted efforts between academic and industry partners to solve these challenges, and further reduce the cost and increase availability will ensure reliability and readiness for clinical use. Finally, integration of analytical techniques with multi-dimensional platforms to obtain high-resolution genomic and proteomic biomarkers can further validate clinical signs of cancer drug-induced cardiotoxicity (Mandawat et al., 2017). Therefore, a multi-dimensional *in vitro* disease modeling approach that ensures reproducibility whilst capturing both acute and chronic effects will offer a boost in the predictive power and development of effective personalized cancer therapies.
